# Infraorbital–Infratrochlear Versus Transnasal Sphenopalatine Ganglion Block for Septorhinoplasty: A Prospective, Randomized, Double-Blind, Clinical Trial

**DOI:** 10.3390/jcm15166321

**Published:** 2026-08-15

**Authors:** Andaç Dedeoğlu, Fatma Acil, Mehmet Özkılıç, Hülya Tosun Soner, Cem Kıvılcım Kaçar

**Affiliations:** Department of Anesthesiology and Reanimation, TR HSU Diyarbakır Gazi Yaşargil TRH, 21070 Diyarbakır, Turkey; anderen77@hotmail.com (A.D.); acilfatma@gmail.com (F.A.); ozkilicmemet@gmail.com (M.Ö.); hulyatosunsoner@hotmail.com (H.T.S.)

**Keywords:** septorhinoplasty, sphenopalatine ganglion block, infraorbital nerve block, infratrochlear nerve block, postoperative pain, emergence agitation

## Abstract

**Background/Objectives**: Regional anesthesia techniques are increasingly being incorporated into multimodal analgesia for septorhinoplasty. This study compared the postoperative analgesic efficacy and recovery profiles of infraorbital–infratrochlear (ION–ITN) nerve block and transnasal sphenopalatine ganglion (SPG) block techniques. **Methods**: In this prospective, randomized, double-blind trial, 93 patients undergoing septorhinoplasty received bilateral ION–ITN block (*n* = 45) or transnasal SPG block (*n* = 48). The primary outcome was postoperative pain assessed using the Numerical Rating Scale (NRS). Peak postoperative NRS scores at the predefined assessment intervals were evaluated with a linear mixed-effects model adjusted for relevant covariates. Continuous variables are presented as medians (first quartile–third quartile [Q1–Q3]), and categorical variables as numbers (percentages). Secondary outcomes included emergence agitation (Riker Sedation–Agitation Scale (RSAS)), rescue tramadol use, patient satisfaction, and adverse events. **Results**: Pain scores decreased significantly over time in both groups (*p* < 0.001). No significant group effect was observed (*p* = 0.726), and adjusted between-group differences were not significant at any postoperative interval (all *p* > 0.05). Although the group-by-time interaction showed a trend toward statistical significance (*p* = 0.053), no meaningful differences in postoperative pain trajectories were observed between the two groups. Median RSAS scores were lower in the SPG group (3 [2,3,4] vs. 4 [3,4], *p* = 0.04), whereas emergence agitation incidence, tramadol use, patient satisfaction, and adverse events were similar. **Conclusions**: Within the multimodal analgesic regimen, no statistically significant differences in postoperative pain outcomes were detected between ION–ITN and transnasal SPG block. Because this study was not designed as an equivalence or non-inferiority trial, these findings should be interpreted as an absence of detected differences rather than proof of equivalent analgesic efficacy. Although SPG block was associated with lower agitation scores, this finding suggests reduced agitation severity rather than superior overall analgesic efficacy.

## 1. Introduction

Septorhinoplasty is one of the most commonly performed facial plastic and functional nasal procedures worldwide and is frequently associated with moderate-to-severe postoperative pain and patient discomfort during the early recovery period [1,2,3]. Although typically performed under general anesthesia in otherwise healthy individuals, inadequate postoperative analgesia may impair the quality of recovery, delay discharge, decrease patient satisfaction, and increase perioperative morbidity [2,3,4,5].

Despite advances in perioperative pain management, postoperative pain after septorhinoplasty remains insufficiently controlled in many patients [6,7]. Furthermore, opioid-based analgesia may be associated with adverse effects, including nausea, vomiting, sedation, respiratory depression, and delayed recovery, thereby increasing interest in opioid-sparing multimodal analgesic strategies [8,9]. Emergence agitation also remains a clinically important concern after septorhinoplasty, with postoperative pain and nasal discomfort considered major contributing factors during emergence from anesthesia [10,11]. Consequently, contemporary perioperative analgesia strategies increasingly support the incorporation of regional anesthesia techniques into multimodal analgesic protocols following septorhinoplasty [1,10,12,13].

Previous clinical studies and meta-analyses have demonstrated that regional nerve block techniques targeting the sensory innervation of the nose and midface have been shown to improve postoperative analgesia, reduce opioid consumption, and decrease emergence agitation following septorhinoplasty [1,4,6,10]. Among these techniques, infraorbital–infratrochlear nerve (ION–ITN) blocks are widely used regional approaches targeting terminal branches of the maxillary and ophthalmic divisions of the trigeminal nerve, thereby providing sensory blockade of the nasal dorsum, septum, nasal tip, and adjacent soft tissues [1,10,14].

Nevertheless, variability in analgesic efficacy has been reported, likely reflecting the complex sensory and autonomic neural pathways involved in septorhinoplasty-related pain [6,15,16]. This has led to growing interest in regional techniques targeting broader autonomic and sensory pathways involved in nasal nociception [7,17,18].

The sphenopalatine ganglion (SPG), located within the pterygopalatine fossa, is a major extracranial parasympathetic ganglion with extensive sensory, sympathetic, and parasympathetic connections involved in nasal innervation and pain transmission [6,15,17,19]. Owing to its central role in nasal sensory and autonomic pathways, SPG blockade has emerged as an important regional analgesic technique in sinonasal surgery and facial pain management [6,7,15,16,17,18]. Several techniques have been described for sphenopalatine ganglion blockade, including minimally invasive transnasal approaches as well as percutaneous techniques that access the pterygopalatine fossa under anatomical or imaging guidance. Although percutaneous approaches allow precise deposition of local anesthetic adjacent to the ganglion, they are technically more demanding and are used primarily for interventional pain management. In contrast, the transnasal approach is simple, minimally invasive, and well suited for routine perioperative analgesia in sinonasal surgery, making it an attractive option for anesthesiologists [20]. Recent randomized trials and systematic reviews suggest that transnasal SPG block may reduce postoperative pain intensity, opioid consumption, and emergence agitation while improving recovery quality and patient satisfaction [1,4,6,10]. Furthermore, this minimally invasive technique is technically straightforward and can be readily incorporated into routine perioperative anesthetic practice [21].

Although recent indirect comparative evidence and network meta-analyses have evaluated these techniques, direct randomized clinical comparisons remain scarce. Because peripheral trigeminal nerve blocks and SPG blockade target different anatomical and nociceptive pathways, their perioperative effects may differ substantially [6,22,23]. Existing studies vary considerably in terms of patient populations, perioperative analgesic protocols, anesthetic strategies, and outcome measures, making direct comparison and interpretation of clinical effectiveness challenging.

Therefore, this randomized, double-blind clinical study aimed to compare the postoperative analgesic efficacy and perioperative clinical outcomes of ION–ITN block and transnasal SPG block in patients undergoing septorhinoplasty under general anesthesia, with the hypothesis that these techniques may demonstrate different effects on postoperative pain and recovery because of their distinct anatomical and physiological mechanisms.

## 2. Materials and Methods

### 2.1. Study Design and Ethics

The study followed a prospective, randomized, double-blind design, and reporting adhered to the Consolidated Standards of Reporting Trials (CONSORT) 2010 statement. The trial was prospectively registered at ClinicalTrials.gov (NCT07345039) on 10 December 2025 before the first participant was enrolled. Ethical approval for the study was obtained from the institutional ethics committee before study initiation. Participant recruitment was conducted between 16 January 2026 and 31 March 2026 in compliance with the 2013 revision of the Declaration of Helsinki. Written informed consent was obtained from every participant prior to study enrollment.

The original protocol prespecified postoperative pain intensity and emergence agitation as co-primary outcomes. Before database lock, any unblinded data access, or formal statistical analysis, the final Statistical Analysis Plan designated postoperative pain intensity as the sole primary endpoint to reduce multiplicity and improve interpretability, whereas emergence agitation was analyzed as a prespecified secondary outcome.

### 2.2. Participants

Patients aged 18–65 years with an American Society of Anesthesiologists (ASA) physical status of I–II, who underwent septorhinoplasty under general anesthesia at TR HSU Diyarbakır Gazi Yaşargil Training and Research Hospital and were able to cooperate with postoperative evaluations, were included in the study.

Patients meeting any of the following criteria were excluded: hypersensitivity or contraindications to local anesthetic agents, coagulopathy or ongoing anticoagulant therapy, infection at the intended injection site, severe nasal anatomical abnormalities precluding transnasal block application, psychiatric or neurological disorders interfering with pain or agitation assessment, chronic pain or chronic opioid use, and refusal to participate.

### 2.3. Randomization and Blinding

Eligible participants were randomly assigned in a 1:1 ratio to either Group 1 (infraorbital–infratrochlear block, ION–ITN) or Group 2 (transnasal sphenopalatine ganglion block, SPG) using a computer-generated random number sequence. Treatment allocation was concealed through the use of sequentially numbered, opaque, sealed envelopes prepared by an independent investigator who had no role in participant recruitment, study interventions, or outcome assessment. These envelopes were opened sequentially immediately before block administration by the anesthesiologist performing the intervention.

Blinding was maintained throughout the study, with both participants and postoperative outcome assessors remaining unaware of treatment allocation. Postoperative evaluations were performed by a blinded anesthesiologist. All block procedures in both study groups were performed by a single experienced anesthesiologist, who was not involved in postoperative outcome assessment, data collection, or statistical analysis. Surgeons and intraoperative care providers were not involved in outcome assessment.

### 2.4. Anesthesia and Perioperative Management

An experienced anesthesiologist performed the preoperative evaluation of all patients in the surgical ward. Patients received detailed preoperative counseling regarding the planned surgical procedure, anesthetic management, and the intended regional block technique for postoperative analgesia. The Numerical Rating Scale (NRS) for postoperative pain assessment was explained to all participants before surgery.

Following a minimum preoperative fasting period of 8 h, patients were transferred to the operating room on the day of surgery. Standard ASA monitoring was applied, including continuous electrocardiography, non-invasive blood pressure measurement, pulse oximetry, and capnographic monitoring.

Venous access was established using an 18–20 G intravenous cannula placed on the dorsum of the hand or in an antecubital vein. An infusion of isotonic 0.9% NaCl (5–10 mL/kg/h) at room temperature was then initiated. All patients received 5 mg intravenous dexamethasone for prophylaxis against postoperative nausea, vomiting, and surgical edema. Preoxygenation was performed with a facemask for three minutes. General anesthesia was induced with intravenous midazolam (0.1 mg/kg) (Vem İlaç, Ankara, Turkey), propofol (2–3 mg/kg) (Fresenius Kabi, Bad Homburg, Germany), fentanyl (2 μg/kg) (Vem İlaç, Ankara, Turkey), and rocuronium (0.6 mg/kg) (Mustafa Nevzat, İstanbul, Turkey). Following adequate neuromuscular relaxation, tracheal intubation was performed using an appropriately sized cuffed endotracheal tube.

After intubation, ventilation was provided with an equal oxygen–air mixture at a fresh gas flow of 3 L/min. Ventilatory parameters were subsequently modified to achieve an end-tidal CO_2_ concentration of 30–35 mmHg. Anesthesia was maintained with sevoflurane (AbbVie, Queenborough, Kent, UK) at 1–2 MAC (end-tidal concentration 1.5–2%), together with a continuous infusion of remifentanil (Vem İlaç, Ankara, Turkey) at 0.05–0.2 μg/kg/min. The remifentanil infusion rate was adjusted to maintain systolic arterial pressure within ±20% of each patient’s baseline value. For surgical antimicrobial prophylaxis, 2 g of intravenous cefazolin (Mustafa Nevzat, İstanbul, Turkey) was administered within 30 min before skin incision. All procedures were conducted using a Dräger Primus anesthesia machine equipped with a Dräger Vapor 2000 sevoflurane vaporizer (Dräger AG, Lübeck, Germany), and intraoperative hemodynamic changes were managed by the attending anesthesiologist.

The nasal septum, turbinates, columella, and nasal dorsum were infiltrated with 10 mL of 1% lidocaine containing epinephrine (10 μg/mL) to provide regional tissue anesthesia and minimize intraoperative blood loss. All patients underwent standardized open septorhinoplasty performed by the same surgical team according to routine institutional practice. Specific surgical maneuvers, including septoplasty, lateral osteotomies, turbinate procedures, nasal valve reconstruction, and additional aesthetic modifications, were performed only when clinically indicated according to each patient’s anatomical characteristics and surgical requirements and were independent of the allocated regional anesthesia technique. Approximately 30 min before the anticipated completion of surgery, all patients received 1 g of intravenous paracetamol (Atabay, İstanbul, Turkey), while 4 mg of intravenous ondansetron (Koçak Farma, İstanbul, Turkey) was administered for prophylaxis against postoperative nausea and vomiting (PONV). Before completion of the procedure, a silicone intranasal splint (Genco Medical Devices, İzmir, Turkey) was positioned for postoperative nasal support. Upon completion of the surgical procedure, neuromuscular blockade was antagonized with intravenous atropine sulfate (0.01 mg/kg) and neostigmine methyl sulfate (0.06 mg/kg) (Adeka, İstanbul, Turkey). The endotracheal tube was removed once patients had regained effective respiratory drive and were able to respond appropriately to spoken instructions. They were subsequently transferred to the post-anesthesia care unit (PACU) for routine monitoring.

Recovery was assessed using the Aldrete score. Patients with an Aldrete score >9 and without significant postanesthetic complications were transferred to the ward. Routine postoperative analgesia consisted of 1 g of intravenous paracetamol administered every 8 h. Postoperative pain was assessed by a physician who remained blinded to treatment allocation. Rescue analgesia was provided with 100 mg of intravenous tramadol when the NRS score was ≥5. If adequate pain relief was not achieved within 30 min, an additional 100 mg dose of tramadol was administered. Patients who developed PONV received 4 mg of intravenous ondansetron as rescue treatment.

### 2.5. Study Interventions

Following induction of general anesthesia and immediately before surgical incision, the assigned regional nerve block was performed. All blocks were carried out by a single experienced anesthesiologist who had no involvement in postoperative outcome assessment, data collection, or statistical analyses. All block procedures were performed under strict sterile conditions.

#### 2.5.1. Bilateral Infraorbital and Infratrochlear Nerve Blocks

Following induction of general anesthesia, patients allocated to Group 1 underwent bilateral infraorbital and infratrochlear nerve blocks performed by a single experienced anesthesiologist. Patients were placed in the supine position with the head maintained in a neutral midline alignment. For the infraorbital nerve block, the infraorbital foramen was identified by palpation of the infraorbital ridge. A 25 G needle (Ayset Inc., Adana, Turkey) was inserted percutaneously through the skin just inferior to the infraorbital foramen and advanced carefully in a cephalad direction toward the infraorbital foramen until the needle tip was palpated beneath the finger placed over the foramen. Care- was taken to avoid entering the infraorbital canal in order to minimize the risk of orbital injury. After negative aspiration, 3 mL of 0.25% bupivacaine (Vem İlaç, Ankara, Turkey) was slowly injected adjacent to the infraorbital foramen after negative aspiration had been confirmed. The infratrochlear nerve block was subsequently performed by inserting the needle 1 cm above the medial canthus and directing it toward the junction of the nasal bone and the medial orbital rim. Following confirmation of negative aspiration, 1 mL of 0.25% bupivacaine was administered. The contralateral side was treated using the same technique and identical volumes of 0.25% bupivacaine. Gentle pressure was applied to each injection site for approximately 1 min to minimize the risk of hematoma formation.

#### 2.5.2. Transnasal Sphenopalatine Ganglion Block

After induction, bilateral transnasal sphenopalatine ganglion block (SPGB) was performed using a cotton-tipped applicator technique under endoscopic guidance by a single experienced anesthesiologist. After topical nasal decongestion with oxymetazoline spray, patients were positioned supine with the head in slight extension to facilitate transnasal access to the nasopharynx. A long cotton-tipped applicator was soaked with 2 mL of 0.25% bupivacaine per side (total 4 mL). The applicator was inserted transnasally along the superior border of the middle turbinate. Under direct endoscopic visualization, it was advanced posteriorly until it reached the mucosa just superior and posterior to the tail of the middle turbinate, corresponding to the sphenopalatine foramen region, where only a thin mucosal layer separates the nasal cavity from the underlying pterygopalatine fossa. Correct applicator placement was confirmed endoscopically before local anesthetic administration. Technical success was defined as successful placement of the applicator in the target region without resistance, significant bleeding, or need for repositioning, with bilateral completion of the planned application protocol. The contralateral side was treated using the same technique and identical volumes of 0.25% bupivacaine. The applicators were positioned bilaterally and left in place for 15 min to ensure adequate transmucosal absorption of the local anesthetic. After the application period, the applicators were removed, and the nasal cavity was examined for bleeding and immediate complications. The transnasal SPG block was completed before local surgical infiltration, surgical preparation of the nasal cavity, and the start of the operative procedure. Following endoscopic confirmation of the absence of clinically significant bleeding or other immediate complications, routine surgical preparation proceeded according to the standard institutional protocol. The transnasal block did not interfere with subsequent septorhinoplasty procedures, including septal, turbinate, and other planned surgical maneuvers.

No block-related technical failures or procedure-related technical complications occurred in either study group. All planned infraorbital–infratrochlear and transnasal sphenopalatine ganglion block procedures were completed successfully without technical failure, significant bleeding requiring applicator repositioning, premature applicator removal, or other procedural difficulties.

### 2.6. Outcome Measures and Data Collection

Preoperatively, all patients’ demographic and physiological characteristics—including age, sex, body mass index (BMI), and ASA physical status—were recorded. The durations of anesthesia and surgery, as well as intraoperative remifentanil consumption, were documented.

The prespecified primary endpoint was postoperative pain intensity at rest, assessed using an 11-point Numerical Rating Scale (NRS; 0 = no pain, 10 = worst imaginable pain). Patients were instructed to rate their overall pain related to the surgical procedure using the NRS. Pain attributable to specific anatomical regions or postoperative conditions (e.g., nasal pain, headache, facial or periorbital pain, or discomfort related to intranasal splints) was not recorded or analyzed separately. NRS assessments at rest were performed hourly during the first 24 postoperative hours by blinded clinical staff. For statistical analysis, the highest NRS value recorded within each predefined postoperative interval (0–2 h, 2–8 h, and 8–24 h after arrival in the PACU) was used to represent that interval, with the intention of capturing clinically relevant peak pain experiences during distinct postoperative phases while reducing variability associated with repeated hourly measurements. Peak interval values were selected a priori to capture clinically meaningful worst pain experiences during distinct postoperative phases while reducing variability associated with repeated hourly measurements. This approach was considered clinically relevant because peak pain intensity is a major determinant of rescue analgesic requirements and patient discomfort in the immediate postoperative period.

Prespecified secondary outcomes included emergence agitation, total tramadol consumption within the first 24 h, patient satisfaction, postoperative nausea and vomiting (PONV), and perioperative adverse events.

Emergence agitation was assessed from the time of extubation until discharge from the PACU using the Riker Sedation–Agitation Scale (RSAS), which scores patient agitation on a seven-point scale ranging from 1 (unarousable) to 7 (dangerously agitated). The highest RSAS score recorded during this period was used for analysis, and a score of ≥5 was considered indicative of emergence agitation (EA).

At 24 h postoperatively, patient satisfaction was assessed using a 5-point Likert scale (1 = very unsatisfied, 2 = quite unsatisfied, 3 = moderate, 4 = quite satisfied, 5 = very satisfied).

Major block-related adverse events, including local anesthetic systemic toxicity, persistent diplopia, clinically significant hematoma, and block failure, were prospectively monitored throughout the perioperative period. Minor procedure-related adverse events, such as self-limited epistaxis or clinically insignificant local bleeding, were not prespecified as separate study outcomes and were therefore not recorded systematically.

### 2.7. Sample Size Calculation

The sample size was calculated a priori using G*Power software (version 3.1.9.4; Heinrich Heine University, Düsseldorf, Germany). The calculation was based on the primary outcome of postoperative pain intensity measured by the Numerical Rating Scale (NRS). According to data from a previous study by Choi et al. [10], mean postoperative NRS scores of 3.00 and 3.67 with standard deviations of 0.78 and 1.56, respectively, were assumed. The sample size was determined assuming a significance level of 0.05 (two-sided), a statistical power of 80%, and a 1:1 allocation ratio. Based on these assumptions, 86 participants (43 in each group) were required to detect an effect size of 0.54. To compensate for anticipated participant attrition, the recruitment target was expanded to 93 patients.

### 2.8. Statistical Analysis

The statistical analyses were prespecified in the final Statistical Analysis Plan (SAP) and performed using Jamovi version 2.5.5 (The Jamovi Project, Sydney, Australia) and R version 4.4.2 (R Foundation for Statistical Computing, Vienna, Austria). Linear mixed-effects models and estimated marginal means were computed in R, while graphical displays were generated using the ggplot2 package (version 3.5.1).

Data distribution was evaluated using the Shapiro–Wilk normality test. Continuous variables exhibiting a normal distribution are reported as mean ± standard deviation (SD), and between-group comparisons were performed with the independent-samples *t*-test. Continuous variables that did not satisfy the assumption of normality are presented as medians (first quartile–third quartile [Q1–Q3]) and were compared using the Mann–Whitney U test. Categorical data are reported as numbers (percentages) and were analyzed using the chi-square test.

Postoperative pain intensity over time was analyzed using the highest hourly Numerical Rating Scale (NRS) value recorded within each predefined postoperative interval (0–2 h, 2–8 h, and 8–24 h) in a covariate-adjusted linear mixed-effects model. The model included group, time, and their interaction (group × time) as fixed effects, with BMI and surgical duration entered as covariates based on their potential clinical relevance to postoperative pain intensity. Time was treated as a categorical repeated-measures factor. A random intercept for each subject was incorporated to account for within-subject correlation due to repeated measurements. Model assumptions (linearity, normality of residuals, and homoscedasticity) were assessed by residual diagnostics.

Estimated marginal means and adjusted between-group differences at each postoperative time interval were calculated from the mixed-effects model. The Holm procedure was used to adjust pairwise comparisons among the estimated marginal means. Treatment effects were reported as adjusted mean differences with corresponding 95% confidence intervals (CIs).

Emergence agitation was defined as a Riker Sedation–Agitation Scale (RSAS) score ≥ 5 and analyzed as a categorical variable. RSAS scores were compared between groups using non-parametric methods due to ordinal scale properties. Given the exploratory nature of RSAS analysis and the limited distribution across categories, non-parametric analysis was considered more robust and clinically interpretable than model-based ordinal approaches.

All statistical analyses were performed using two-sided tests, and a *p*-value < 0.05 was considered statistically significant.

## 3. Results

### 3.1. Patient Flow and Baseline Characteristics

**Figure 1:**
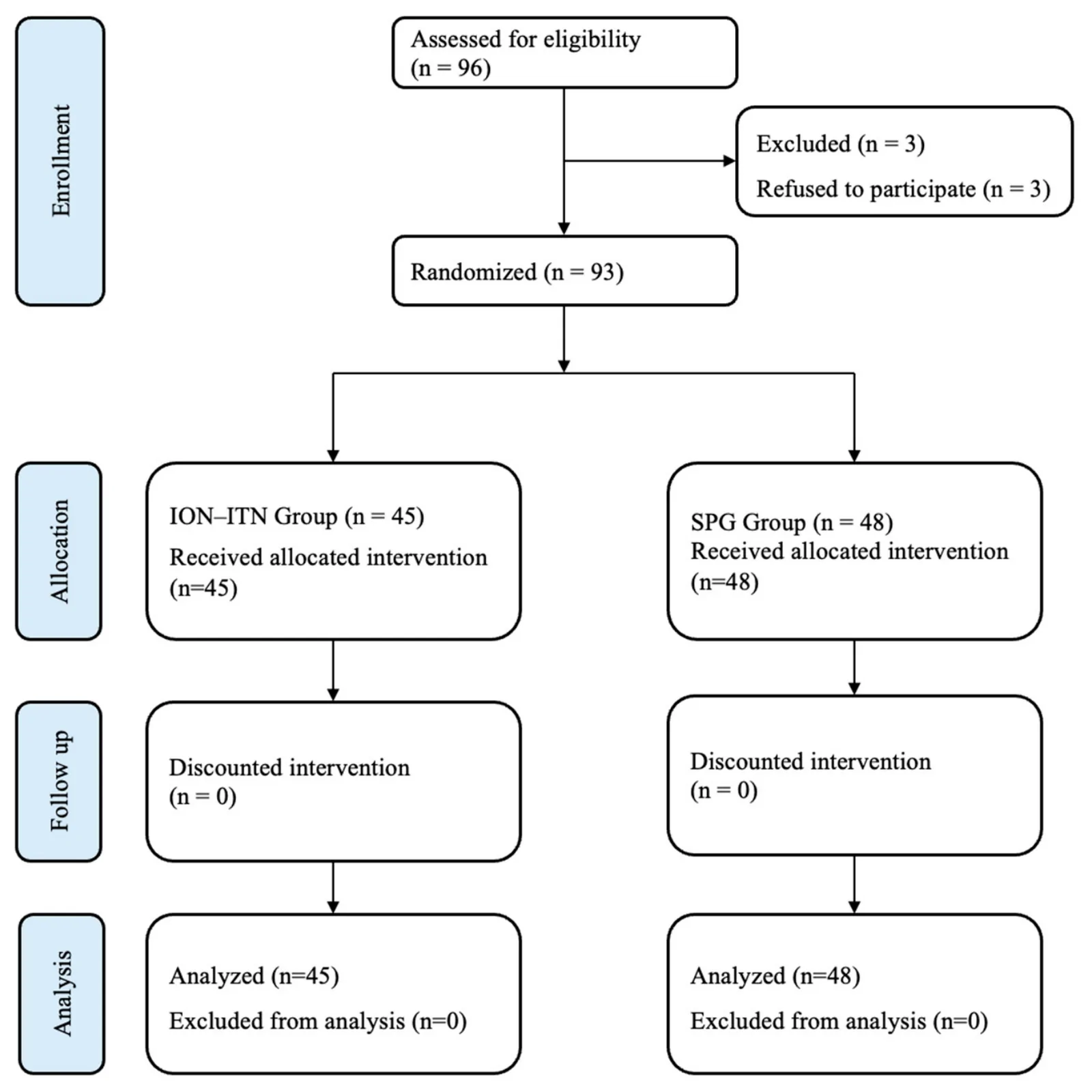
CONSORT flow diagram showing patient enrollment, randomization, follow-up, and analysis.

A total of 96 patients were screened for eligibility. Three patients declined participation and were excluded prior to randomization. Consequently, 93 patients were randomized: 45 were allocated to the infraorbital–infratrochlear nerve block group (ION–ITN) and 48 to the sphenopalatine ganglion block group (SPG). All randomized participants underwent the assigned intervention and completed all study procedures. None were lost during follow-up, and all randomized participants were included in the final analysis. The CONSORT flow diagram is presented in Figure 1.

Baseline demographic and perioperative characteristics are summarized in Table 1. No significant between-group differences were observed in age, sex distribution, ASA physical status, anesthesia duration, or intraoperative remifentanil consumption (all *p* > 0.05). Body mass index (BMI) was significantly higher in the SPG group compared with the ION–ITN group (median [Q1–Q3]: 29.44 [27.42–31.30] vs. 27.68 [26.59–29.29] kg/m^2^, *p* = 0.018). Surgical duration was modestly but statistically significantly longer in the ION–ITN group (55.9 ± 3.7 vs. 53.7 ± 6.1 min, *p* = 0.041). These variables were therefore included as covariates in the adjusted analyses owing to their potential influence on postoperative pain.

### 3.2. Primary Outcome

Postoperative pain intensity over time was evaluated using a covariate-adjusted linear mixed-effects model that included group, time, BMI (z-score), surgical duration (z-score), and a random intercept for subjects (Table 2).

Peak postoperative NRS scores decreased significantly over time (F = 52.95, *p* < 0.001). Group allocation was not significantly associated with postoperative pain intensity (*p* = 0.726). The group × time interaction approached, but did not reach, statistical significance (*p* = 0.053). However, none of the adjusted interval-specific comparisons reached statistical significance (all *p* > 0.05). Therefore, the observed interaction should be interpreted cautiously and may represent subtle temporal differences that require confirmation in adequately powered studies.

**Figure 2:**
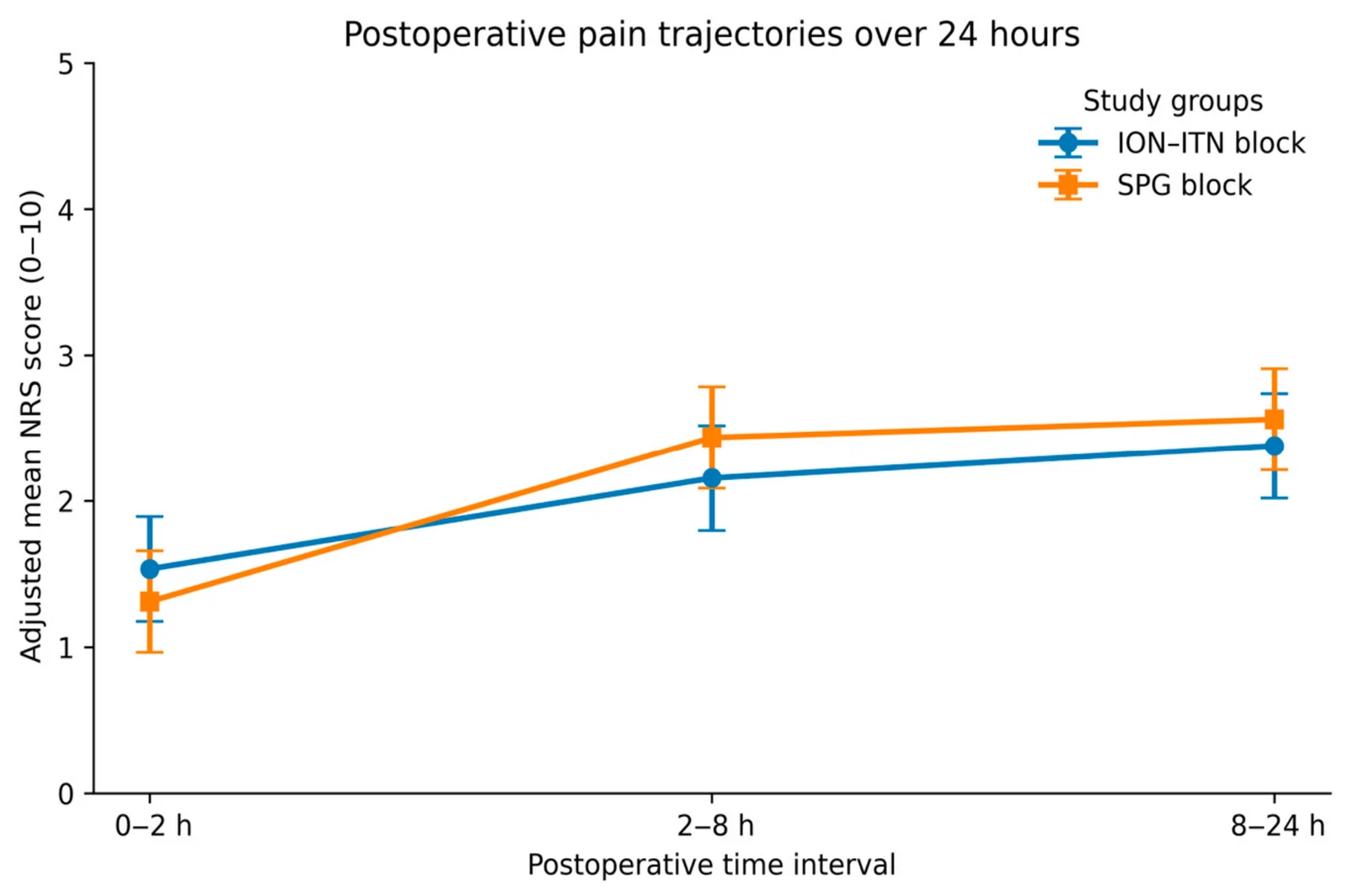
Adjusted postoperative pain trajectories over 24 h. Adjusted mean peak Numerical Rating Scale (NRS) scores representing the highest hourly NRS value recorded within each predefined postoperative interval (0–2 h, 2–8 h, and 8–24 h) for the infraorbital–infratrochlear (ION–ITN) and sphenopalatine ganglion (SPG) block groups. Values represent estimated marginal means derived from a covariate-adjusted linear mixed-effects model. Error bars indicate 95% confidence intervals. Both groups showed progressive reductions in pain intensity over time.

Adjusted between-group differences in postoperative NRS scores at each predefined time interval are presented in Table 3 and Figure 2. Adjusted mean differences were small across all postoperative intervals, with all corresponding 95% confidence intervals spanning zero. None of the interval-specific comparisons reached statistical significance (all *p* > 0.05).

### 3.3. Secondary Outcomes

Postoperative agitation and recovery-related outcomes are presented in Table 4.

**Figure 3:**
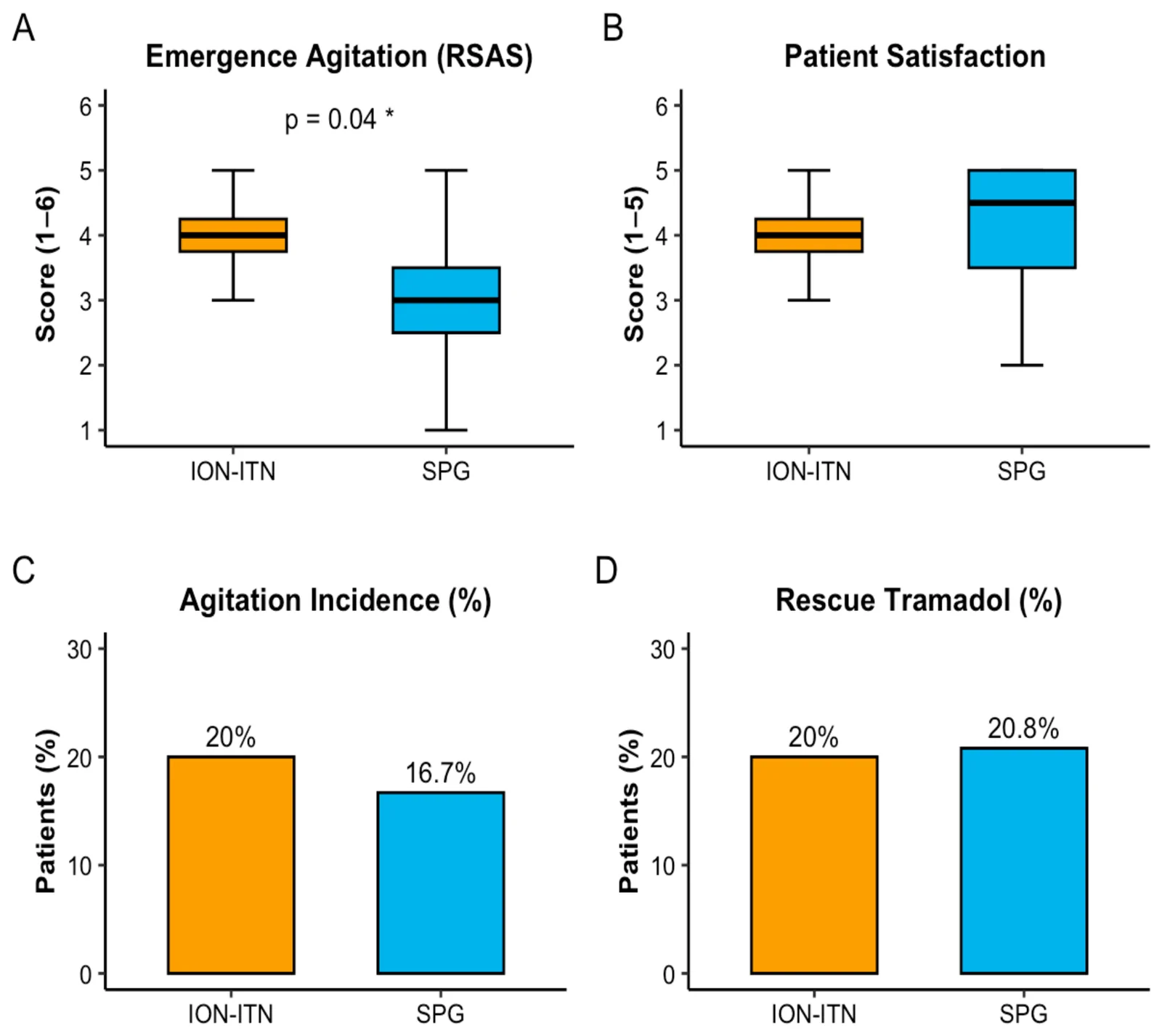
Comparison of clinical outcomes: (**A**) Riker Sedation–Agitation Scale (RSAS) scores at emergence; the sphenopalatine ganglion (SPG) group demonstrated significantly lower scores compared to the infraorbital–infratrochlear (ION–ITN) group (*p* = 0.04). (**B**) Postoperative patient satisfaction scores. (**C**) Incidence of emergence agitation (RSAS ≥ 5). (**D**) Requirement for rescue tramadol analgesia within the first 24 h. Lower RSAS scores in the SPG group reflect reduced agitation severity rather than a lower incidence of emergence agitation. * *p* < 0.05 indicates statistical significance.

Median Riker Sedation–Agitation Scale (RSAS) scores differed between the two groups. The ION–ITN group had a median [Q1–Q3] of 4 [3,4], whereas the SPG group had a median [Q1–Q3] of 3 [2,3,4] (*p* = 0.04), indicating greater agitation during the early recovery period (Figure 3A). In contrast, the incidence of emergence agitation, defined as RSAS ≥ 5, did not differ significantly between groups (20.0% in the ION–ITN group vs. 16.7% in the SPG group, *p* = 0.68; Figure 3C).

Patient satisfaction at 24 h was similar between groups (*p* = 0.12; Figure 3B). Overall tramadol consumption was similar between groups (*p* = 0.91; Table 4). The proportion of patients requiring rescue tramadol was also similar between groups (20.0% vs. 20.8%, *p* = 0.96; Figure 3D).

No major block-related adverse events occurred in either group. No cases of hematoma, diplopia, local anesthetic systemic toxicity, clinically significant epistaxis, block-related neurological complications, or block failures requiring repeat intervention were observed.

## 4. Discussion

In this prospective, randomized, double-blind clinical trial, we compared the postoperative analgesic and recovery profiles of bilateral infraorbital–infratrochlear (ION–ITN) nerve block and transnasal sphenopalatine ganglion (SPG) block in patients undergoing septorhinoplasty. This study yielded three principal findings. First, no statistically significant differences in postoperative analgesic outcomes were detected between the ION–ITN and SPG block techniques. Second, SPG block was associated with lower early postoperative agitation scores despite a similar incidence of emergence agitation. Third, opioid consumption and patient satisfaction were similar between groups, supporting the clinical utility of either technique as part of multimodal analgesia.

A key finding of this study is that both techniques yielded similar postoperative pain scores. This finding should be interpreted within the context of the comprehensive multimodal analgesic regimen used in the present study, which included local surgical infiltration with lidocaine and epinephrine, intraoperative remifentanil infusion, and scheduled postoperative paracetamol in addition to the allocated regional nerve block. This standardized multimodal analgesic strategy may have reduced the ability to detect small differences in analgesic efficacy between the two regional techniques. Nevertheless, the lack of a statistically significant difference does not justify concluding that the two interventions are equivalent. The present trial was neither designed nor adequately powered to evaluate equivalence or non-inferiority, and therefore small but clinically important differences cannot be ruled out. Consequently, these findings should be viewed as indicating that no statistically demonstrable difference was identified under the conditions of this study, rather than establishing equivalent clinical performance. Previous studies have consistently demonstrated that each technique, when compared with control groups, significantly reduces postoperative pain and opioid consumption. The combined ION–ITN block has been shown to decrease pain scores, opioid requirements, and recovery unit stay [1] while also reducing emergence agitation and improving analgesic quality [4,10]. Similarly, SPG block has been associated with lower postoperative pain scores and reduced analgesic consumption in nasal surgery [24,25].

Unlike prior studies, which primarily compared individual regional techniques with placebo or no-block control groups, the present study was specifically designed as an active-comparator trial evaluating two clinically established regional anesthesia techniques under standardized perioperative conditions. Consequently, our study was designed to compare the relative clinical performance of these two active regional analgesic strategies rather than to determine the absolute benefit of either technique compared with standard multimodal analgesia without regional blockade. Recent network meta-analytic evidence has attempted indirect comparisons between SPG and infraorbital–infratrochlear techniques. Albilasi et al. recently performed a systematic review and network meta-analysis evaluating the efficacy and safety of SPG and infraorbital–infratrochlear blocks in nasal and sinus surgeries [22]. However, such analyses remain dependent on between-study assumptions and heterogeneous perioperative protocols. Our study directly compared these techniques under standardized perioperative conditions in a randomized clinical trial.

The comprehensive multimodal analgesic regimen, including local surgical infiltration, systemic analgesics, and regional anesthesia, likely contributed to uniformly low postoperative pain scores and opioid consumption in both groups. Under these conditions, any true differences between the two regional techniques may have been difficult to detect. Accordingly, the absence of statistically significant between-group differences may reflect a ceiling effect of analgesia, whereby both approaches sufficiently suppressed nociceptive input from the nasal and septal structures. This finding was further supported by the modest between-group effect estimates, with confidence intervals consistently including the null value throughout the postoperative assessments. Although the group-by-time interaction showed a trend toward statistical significance, these findings should be interpreted cautiously, as temporal differences between the interventions cannot be excluded.

The similar analgesic outcomes observed may also be explained by the complementary mechanisms of the two techniques. ION–ITN block primarily provides peripheral somatic blockade of trigeminal nerve branches innervating the nasal dorsum and septum. In contrast, SPG block acts at the level of the pterygopalatine fossa, influencing not only sensory transmission via the maxillary nerve but also parasympathetic pathways involved in vasodilation and neurogenic inflammation and trigeminal–autonomic reflexes. The SPG plays a well-established role in trigeminal–autonomic modulation and has been widely utilized in the management of various facial pain and headache disorders [16,26].

Recent anatomical evidence has further clarified the mechanism underlying transnasal SPG blockade. A cadaveric study by Istenič et al. demonstrated that topically applied bupivacaine at the level of the sphenopalatine foramen diffuses across the thin mucosal layer into the pterygopalatine fossa, with drug concentrations decreasing exponentially as the distance from the sphenopalatine foramen increases, supporting passive diffusion as the primary transport mechanism. These findings also emphasize that effective transnasal SPG blockade depends on accurate applicator placement at the sphenopalatine foramen, sufficient contact time to facilitate transmucosal diffusion, and an appropriate local anesthetic concentration. In our study, the applicator was positioned under direct endoscopic visualization at the anatomical region of the sphenopalatine foramen and maintained in place for 15 min, closely reflecting these evidence-based technical principles and thereby strengthening the methodological validity of the transnasal SPG block protocol used in our study [27].

Despite these anatomical and physiological differences, both techniques ultimately reduce afferent nociceptive signaling from the surgical field, which may explain the similar pain outcomes observed. Furthermore, the established role of SPG blockade in chronic facial pain and headache syndromes supports its function as a broader neuromodulatory target.

An important and clinically relevant finding is the dissociation between agitation severity and incidence. Although the incidence of emergence agitation did not differ between groups, RSAS scores were significantly lower in the SPG group. These findings may suggest attenuation of agitation severity rather than a reduction in the occurrence of emergence agitation itself. Accordingly, the observed difference in RSAS should be interpreted as reflecting a difference in agitation severity rather than a clinically meaningful reduction in the incidence of emergence agitation.

Emergence agitation is multifactorial but strongly associated with postoperative pain and airway stimulation [10]. In our study, given that pain scores were similarly low in both groups and no statistically significant differences were observed in postoperative pain outcomes, the lower agitation severity observed in the SPG group may reflect reduced autonomic stimulation, attenuation of postoperative nasal discomfort and congestion, and modulation of trigeminal–autonomic pathways related to SPG blockade; however, these potential mechanisms remain speculative. The SPG plays a key role in parasympathetic regulation and trigeminal–autonomic reflexes, and its blockade may therefore contribute to smoother emergence characteristics through autonomic modulation and reduction in postoperative nasal sensory input. However, because detailed autonomic function and perioperative hemodynamic parameters were not evaluated, no conclusions regarding perioperative hemodynamic stability can be drawn.

Another notable finding was the very low postoperative opioid requirement in both groups, with no significant difference between them. This finding further supports the effectiveness of combining regional anesthesia with a multimodal analgesic regimen. The near-zero tramadol consumption suggests a floor effect for rescue opioid use, which may have limited the ability to detect small differences between the two techniques. Future studies incorporating more sensitive outcome measures or higher-risk patient populations may better delineate potential differences in analgesic efficacy.

Patient satisfaction was similarly high in both groups, reinforcing that both techniques provided clinically meaningful postoperative analgesia. Together with the comparable pain scores and minimal opioid requirements, these findings suggest that either block can be effectively incorporated into multimodal analgesia protocols for septorhinoplasty. These observations are consistent with previous clinical studies reporting improved patient satisfaction and reduced analgesic requirements following both ION–ITN and SPG blocks [4,24].

Clinically, these results indicate that ION–ITN and SPG blocks are effective components of multimodal analgesia in septorhinoplasty. Given their comparable postoperative analgesic performance, the choice between techniques may reasonably depend on practical considerations such as operator experience, ease of application, invasiveness, resource availability, patient-specific anatomical characteristics, contraindications, and the respective procedural risk profiles. For example, transnasal SPG block may be less suitable in patients with severe nasal anatomical abnormalities, active nasal bleeding, or conditions limiting transnasal access, whereas ION–ITN block may be preferable in such circumstances. Conversely, transnasal SPG block may be advantageous when avoidance of multiple facial needle punctures is desired. Therefore, selection of the regional anesthesia technique should be individualized according to the clinical context and patient characteristics. The lower agitation scores observed with SPG block may represent an additional advantage in patients at increased risk of emergence-related complications. Furthermore, no major block-related adverse events occurred in either group. These findings suggest that regional block techniques can be performed safely by experienced anesthesiologists.

Although no allergic or hypersensitivity reactions were observed in the present study, perioperative anaphylaxis remains a rare but potentially life-threatening complication associated with anesthetic and perioperative medications. Therefore, careful preoperative assessment of allergy history, preparedness for the prompt recognition and management of hypersensitivity reactions, and vigilance for their early detection remain essential. Emerging approaches, including artificial intelligence-assisted perioperative risk prediction, may further enhance perioperative patient safety in the future [28].

Although the total injected volumes differed between the two block techniques, the administered volumes were determined according to the anatomical requirements and commonly described technical approaches of each procedure. The ION–ITN block requires broader peripheral spread, whereas the SPG block targets a relatively confined anatomical space adjacent to the ganglion. Importantly, the same local anesthetic concentration was used in both groups. Nevertheless, differences in local anesthetic volume may have influenced the extent of anesthetic spread and should therefore be considered a potential source of confounding when interpreting the findings. In addition, accumulating anatomical evidence suggests that the route of drug administration is another important determinant of injectate distribution within the pterygopalatine fossa. Different approaches, including transnasal, transoral, and posterior infrazygomatic techniques, produce distinct injectate distribution patterns despite similar injectate volumes, which may influence the extent of ganglion exposure and, consequently, clinical outcomes. Therefore, both injectate volume and the route of administration should be considered when interpreting and comparing the efficacy of different sphenopalatine ganglion block techniques [29].

Several limitations should be acknowledged. First, because no placebo or no-block control group was included, the study was designed as an active-comparator trial and therefore cannot determine the absolute benefit of either regional anesthesia technique relative to standard multimodal analgesia without regional blockade. In addition, this was a single-center study, and all regional blocks were performed by a single experienced anesthesiologist. Although this approach enhanced procedural standardization and minimized technical variability, it may limit the generalizability of the findings to institutions with different patient populations, clinical practices, and levels of operator experience. Second, the low postoperative tramadol requirement observed in both groups, while clinically favorable and consistent with the effectiveness of the standardized multimodal analgesic regimen, may have limited the ability to detect small differences in analgesic efficacy between the two regional anesthesia techniques, particularly with respect to rescue opioid consumption. Furthermore, all patients received standardized surgical infiltration with lidocaine and epinephrine into the nasal septum, concha, columella, and dorsum as part of the routine septorhinoplasty procedure. Although this approach reflects standard clinical practice and was applied equally in both groups, it may have further reduced early postoperative pain intensity and attenuated potential differences in analgesic efficacy between the two regional anesthesia techniques. Third, although BMI and surgical duration were adjusted in the statistical model, baseline imbalances may still have influenced outcomes. Additionally, although the statistical analysis plan was finalized prior to database locking and before any unblinded analysis, modification of the prespecified outcome hierarchy from co-primary outcomes to a single primary endpoint may still be considered a potential source of bias. Another limitation is that block performance time was not prospectively recorded. Procedural efficiency is an important practical consideration when comparing regional anesthesia techniques and may influence their implementation in routine clinical practice. Future comparative studies should therefore evaluate block performance time alongside clinical efficacy, safety, and patient-centered outcomes. Another limitation is that objective confirmation of successful blockade by preoperative sensory testing was not feasible because both regional block techniques were performed after induction of general anesthesia. Consequently, block success was inferred from standardized block administration and subsequent postoperative clinical outcomes rather than direct sensory assessment. Future studies in which regional blocks are performed before induction of general anesthesia should incorporate objective sensory testing to confirm successful blockade. In addition, although major block-related complications were monitored throughout the study, minor procedure-related adverse events, such as self-limited epistaxis or clinically insignificant local bleeding, were not prospectively recorded as separate study outcomes. Future studies should incorporate standardized reporting of both major and minor adverse events to provide a more comprehensive evaluation of the safety profile of these regional anesthesia techniques.

Furthermore, although postoperative pain assessments were performed hourly, analysis based on the highest NRS value within relatively broad postoperative intervals may have reduced sensitivity for detecting short-lived temporal differences in analgesic efficacy, particularly during the immediate postoperative period. Likewise, the use of interval-based peak NRS values rather than all repeated hourly observations decreased temporal resolution and may have limited statistical power to detect transient analgesic differences. In addition, postoperative pain was assessed as overall pain related to the surgical procedure using a single global NRS, and pain attributable to specific anatomical regions or postoperative conditions (e.g., nasal pain, headache, facial or periorbital pain, or discomfort related to the intranasal splints) was not recorded or analyzed separately. Consequently, potential differential effects of the two regional techniques on specific components of postoperative pain could not be determined.

Finally, although the group × time interaction approached statistical significance, the study may have been underpowered to detect subtle temporal differences. Moreover, RSAS scores were analyzed using non-parametric methods; future studies employing ordinal regression models may provide a more refined evaluation of emergence-related behavioral outcomes. Perioperative hemodynamic parameters were also not formally analyzed; therefore, the potential influence of the regional techniques on hemodynamic stability remains uncertain.

## 5. Conclusions

In this randomized comparison of two active regional techniques within a multimodal analgesic regimen, no statistically significant differences were observed in postoperative pain outcomes. Rather than demonstrating the superiority of one technique over the other, our findings support the clinical use of either technique within a standardized multimodal analgesic regimen. Both groups were associated with similarly low opioid consumption and high patient satisfaction. Although SPG block was associated with lower early postoperative agitation scores, this finding suggests a potential advantage in recovery characteristics rather than superior overall analgesic efficacy. In routine clinical practice, the choice between ION–ITN and SPG block should be guided by operator expertise, technical feasibility, patient-specific anatomical characteristics, the risk of nasal bleeding, and institutional preferences rather than by the expectation of superior analgesic efficacy.

## Figures and Tables

**Table 1:** Demographic and perioperative characteristics of the study groups.

Variable	ION–ITN Block (*n* = 45)	SPG Block (*n* = 48)	*p*-Value
Age (years)	42.7 ± 9.4	44.9 ± 8.8	0.245
Sex			0.665
Female	30 (67%)	34 (70%)	
Male	15 (33%)	14 (30%)	
BMI (kg/m^2^)	27.68 (26.59–29.29)	29.44 (27.42–31.30)	0.018 *
ASA Class			0.378
I	18 (40%)	15 (31%)	
II	27 (60%)	33 (69%)	
Surgical duration (min)	55.9 ± 3.7	53.7 ± 6.1	0.041 *
Anesthesia duration (min)	73 (70–75)	70 (70–75.8)	0.575
Intraoperative remifentanil consumption (µg·kg^−1^·min^−1^)	0.30 ± 0.18	0.29 ± 0.16	0.599

Values are presented as mean ± SD or median (first quartile–third quartile [Q1–Q3]), as appropriate. BMI: body mass index; ASA: American Society of Anesthesiologists; ION–ITN: infraorbital–infratrochlear nerve block; SPG: sphenopalatine ganglion block. * *p* < 0.05 indicates statistical significance.

**Table 2:** Covariate-adjusted linear mixed-effects model for postoperative NRS.

Effect	NumDF	DenDF	F	*p*-Value
Group	1	89	0.1232	0.726
Time	2	182	52.9499	<0.001 *
BMI	1	89	0.9767	0.326
SurgDur	1	89	0.7552	0.387
Group × Time	2	182	2.9887	0.053

BMI, body mass index; DenDF, denominator degrees of freedom; NumDF, numerator degrees of freedom; NRS, numerical rating scale; SurgDur, surgical duration. * *p* < 0.05 indicates statistical significance.

**Table 3:** Adjusted between-group differences in peak postoperative NRS scores at each time interval (estimated marginal means).

Time Interval	ΔNRS (Group1–Group2)	95% CI	*p*-Value
0–2 h	−0.22	−0.73 to 0.29	0.388
2–8 h	0.28	−0.23 to 0.79	0.282
8–24 h	0.18	−0.33 to 0.69	0.482

ΔNRS represents the difference in mean Numerical Rating Scale scores between the ION–ITN and SPG groups. CI, confidence interval. *p* < 0.05 indicates statistical significance.

**Table 4:** Emergence agitation, patient satisfaction, and analgesic consumption.

Variable	ION–ITN Block (*n* = 45)	SPG Block (*n* = 48)	*p*-Value
RSAS score	4 (3–4)	3 (2–4)	0.04 *
Emergence agitation (RSAS ≥ 5)	9 (20%)	8 (16.7%)	0.68
Patient satisfaction score	4 (3–4)	4 (3–5)	0.12
Rescue tramadol requirement	9 (20%)	10 (20.8%)	0.96
Total tramadol consumption (mg)	0 (0–0)	0 (0–0)	0.91

Values are presented as median (Q1–Q3) or number (%), as appropriate. Tramadol requirement is presented as the number of patients requiring at least one dose of rescue analgesia. ION–ITN, infraorbital–infratrochlear nerve block; SPG, sphenopalatine ganglion block; RSAS, Riker Sedation–Agitation Scale; Q1–Q3, first quartile–third quartile. * *p* < 0.05 indicates statistical significance.

## Data Availability

The data presented in this study are available from the corresponding author upon reasonable request. The data are not publicly available because they contain de-identified individual participant data derived from a clinical trial, and unrestricted public sharing was not covered by the ethics committee approval. Access to the data may be granted for scientific research purposes, subject to institutional ethical requirements and applicable data protection regulations.

## Abbreviations

The following abbreviations are used in this manuscript:

BMI	Body Mass Index
NRS	Numerical Rating Scale
RSAS	Riker Sedation–Agitation Scale
SPG	Sphenopalatine Ganglion
ION–ITN	Infraorbital–Infratrochlear Nerve
ASA	American Society of Anesthesiologists
SD	Standard Deviation
IQR	Interquartile Range
